# The hemicellulolytic enzyme arsenal of *Thermobacillus xylanilyticus* depends on the composition of biomass used for growth

**DOI:** 10.1186/1475-2859-11-159

**Published:** 2012-12-14

**Authors:** Harivony Rakotoarivonina, Béatrice Hermant, Nina Monthe, Caroline Rémond

**Affiliations:** 1 UMR FARE614 Université de Reims Champagne-Ardenne, Reims, France; 2 UMR FARE614 INRA, Reims, France

**Keywords:** *Thermobacillus xylanilyticus*, Hemicellulases production, Growth, Wheat bran, Wheat straw

## Abstract

**Background:**

*Thermobacillus xylanilyticus* is a thermophilic and highly xylanolytic bacterium. It produces robust and stable enzymes, including glycoside hydrolases and esterases, which are of special interest for the development of integrated biorefineries. To investigate the strategies used by *T. xylanilyticus* to fractionate plant cell walls, two agricultural by-products, wheat bran and straw (which differ in their chemical composition and tissue organization), were used in this study and compared with glucose and xylans. The ability of *T. xylanilyticus* to grow on these substrates was studied. When the bacteria used lignocellulosic biomass, the production of enzymes was evaluated and correlated with the initial composition of the biomass, as well as with the evolution of any residues during growth.

**Results:**

Our results showed that *T. xylanilyticus* is not only able to use glucose and xylans as primary carbon sources but can also use wheat bran and straw. The chemical compositions of both lignocellulosic substrates were modified by *T. xylanilyticus* after growth. The bacteria were able to consume 49% and 20% of the total carbohydrates in bran and straw, respectively, after 24 h of growth. The phenolic and acetyl ester contents of these substrates were also altered. Bacterial growth on both lignocellulosic biomasses induced hemicellulolytic enzyme production, and xylanase was the primary enzyme secreted. Debranching activities were differentially produced, as esterase activities were more important to bacterial cultures grown on wheat straw; arabinofuranosidase production was significantly higher in bacterial cultures grown on wheat bran.

**Conclusion:**

This study provides insight into the ability of *T. xylanilyticus* to grow on abundant agricultural by-products, which are inexpensive carbon sources for enzyme production. The composition of the biomass upon which the bacteria grew influenced their growth, and differences in the biomass provided resulted in dissimilar enzyme production profiles. These results indicate the importance of using different biomass sources to encourage the production of specific enzymes.

## Background

The development of biorefineries represents a key advance in access to the integrated production of bio-derived products, such as energy (fuels, heat), chemicals and materials [1]. Various starting materials, including agricultural residues (such as sugarcane bagasse, corn stover, wheat bran (WB) and wheat straw (WS)) and forest residues, represent biomass substrates of interest to biorefineries [2,3]. The development of integrated biorefineries requires valorizing the entire plant, and in this context, the transformation of hemicellulosic components of plant cell walls offers new opportunities for biorefineries to produce high value molecules, such as alkyl pentosides [4,5].

Lignocellulosic plant cell walls are an assembly of cellulose, lignin and hemicelluloses. These polymers are linked together by covalent and non-covalent linkages and form an organized network. Cellulose, main polysaccharide in the plant cell wall, represents 35% to 50% of the dry matter of cell walls [6]. Hemicelluloses represent 25% to 50% of the dry matter in plant cell walls and are heteropolysaccharides with compositions that vary according to their plant origins. Arabino-glucurono-xylans are the most abundant hemicelluloses found in graminaceous plants. They are formed by linear chains of xylans that comprise β-(1,4)-linked D-xylopyranose residues. These chains can contain various substitute residues, such as L-arabinofuranose, glucuronic and 4-*O*-methyl-glucuronic acids, as well as other acetyl groups. In graminaceous plant cell walls, arabinose residues can be esterified by phenolic compounds, such as ferulic or *p*-coumaric acids. Ferulic acid can form diferulic bridges that link two xylan chains together or associate hemicelluloses to lignin [7,8]. Lignin is a complex phenolic polymer responsible for the rigidity and impermeability of the plant cell wall, and it represents 10% to 35% of the dry matter in plant cell walls [6].

The fractionation of lignocellulosic plant cell walls requires the development of efficient technologies because the complex structures of plant cell walls hinder the extraction and fractionation of their structural polysaccharides. Several chemical and physical treatments are able to solubilize and hydrolyze cellulose and hemicelluloses [9], but the use of biological transformations (including the use of microorganisms and enzymes) is more beneficial in terms of their energy savings, specificity of action and environmental friendliness.

In nature, lignocellulosic biomass degradation is carried out by microorganisms (bacteria, fungi and protozoa) found in various natural ecosystems such as in the digestive tracts of animals, in soils and in water. Cooperative action occurs between different microorganisms, allowing for the complete degradation of plant cell walls [10-12]. These microorganisms are able to efficiently degrade cellulose, hemicelluloses and lignin with the goal of allowing the production of monomeric molecules for use as carbon sources for growth or for secondary metabolite production. Because lignocellulosic biomasses are recalcitrant starting materials, their degradation imposes several challenges for microorganisms, notably the production of a panel of various enzymes to fractionate polymers constitutive of lignocellulosic biomass. These enzymes are mainly glycoside hydrolases (cellulases, hemicellulases) and esterases, as well as lignolytic enzymes produced by some fungi. In the case of the glycoside hydrolases, cooperative action between endo-enzymes and exo-acting enzymes is required for the liberation of monosaccharides [13].

One method for developing enzymatic cocktails for the optimal fractionation of polysaccharides is to gain a better understanding of the dynamic strategies used by the microorganisms that are able to fractionate polysaccharides and apply this knowledge to the design of biotechnological processes. Hemicellulases play an important role in efficient hydrolysis, as these enzymes can be used to create better access to other enzymes within the cellulose fibrils that are embedded in plant biomass [14,15]. *T. xylanilyticus* is an aerobic, gram-positive, thermophilic and hemicellulolytic bacterium with no cellulase activity [16,17]. Four thermostable enzymes have been obtained and characterized from this bacterium: two xylanases, one arabinofuranosidase and one feruloyl esterase [18-22]. These purified enzymes are able to efficiently degrade plant cell walls, such as the cell walls of WB and WS [22-26].

The aim of this work was to investigate strategies used by *T. xylanilyticus* to fractionate wheat bran and straw, two agricultural by-products that are different in terms of their chemical compositions and tissue organization. The ability of *T. xylanilyticus* to grow on both of these substrates was studied and compared to its growth on classical substrates (glucose and xylans). Hemicellulolytic enzyme production was evaluated and correlated with initial biomass composition, as well as the composition of the residues produced during growth.

## Results

### *T. xylanilyticus* growth kinetics on various substrates

**Figure 1:**
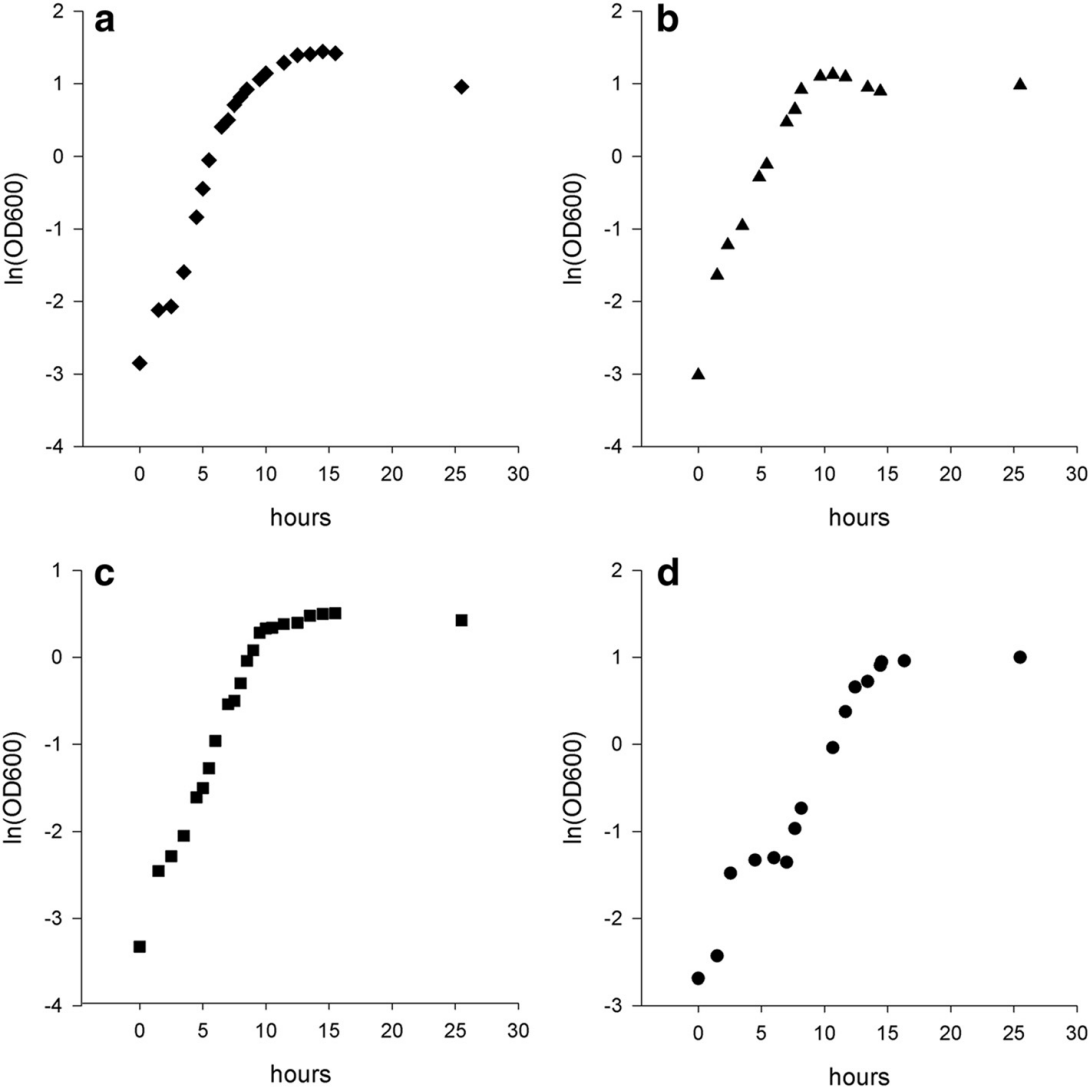
**Growth curves of *T. xylanilyticus *cultivated on various substrates.** Growth was expressed as the ln of absorbance at 600 nm as a function of incubation time. (**a**), (**b**), (**c**) and (**d**) show the kinetic curves obtained with glucose, xylan, wheat bran and wheat straw cultures, respectively. The results are mean values of triplicates, and standard deviations are less than 5%.

**Figure 2:**
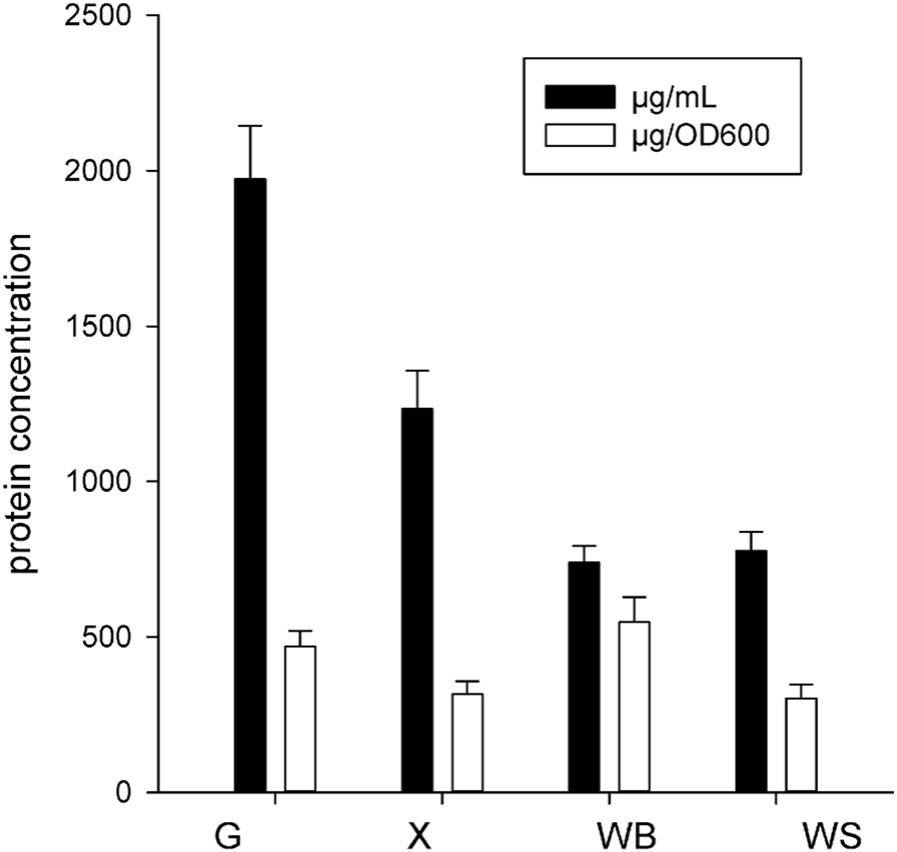
**Total protein concentrations obtained with *T. xylanilyticus *growing on various substrates during the stationary phase.** Black bars represent protein concentrations in μg/mL. White bars represent protein concentrations correlated to cell densities. The results are the mean values of the samples performed in triplicate. G, glucose; X, xylan; WB, wheat bran; and WS, wheat straw.

Growth profiles were determined for three different cultures that included monosaccharide (glucose), polysaccharides (xylan) and two different types of plant cell walls (WB and WS) by monitoring turbidity of the culture medium for 25 h. Figure 1 shows the growth profiles of *T. xylanilyticus* based on the various substrates tested. *T. xylanilyticus* was able to use glucose, xylan and lignocellulosic biomasses as primary carbon sources for energy production. Growth was rapid on glucose, with a generation time of 111 ± 2 min. On xylan and WB, *T. xylanilyticus* had comparable generation times (107 ± 9 min and 104 ± 3 min, respectively). However, its growth on WB showed a significant increase, as its doubling time was higher compared to that observed for glucose (p < 0.05). *T. xylanilyticus* was able to use WS as a substrate but did not use it as efficiently as other substrates. The doubling time on WS was significantly higher (132 ± 4 min) (p < 0.05) than on other substrates. Growth kinetics curves on glucose, xylan and WB exhibited similar profiles (Figure 1a-b-c). Exponential phases were reached quickly, either without or after a very short lag phase, and the stationary phase was defined as an OD_600 nm_ greater than 3 for glucose and xylan and an OD_600 nm_ greater than 1.3 for WB. During the stationary phase, sporulation occurred rapidly in xylan and glucose cultures (the turbidity of the medium decreased dramatically), whereas no or few sporulated cells were observed in the WB culture after 13 h. On WS, the *T. xylanilyticus* growth profile was again different, following a kinetic curve with two successive growths. During the first step, absorbance of the culture at 600 nm increased to 0.23. This step was followed by a long lag phase (4.5 h) and new acceleration and exponential phases (Figure 1d). The generation time was calculated during the second exponential phase. The stationary phase was defined as an OD_600 nm_ of greater than 2.5 on WS. Glucose seemed to be a better substrate for use in obtaining a high quantity of bacterial biomass, as absorbance of the culture on glucose was approximately 4.2 at the stationary phase. This result corresponded with a cell concentration of 1.8 × 10^9^ cells/mL. On lignocellulosic substrates, the number of cells obtained was lower (6.1 × 10^8^ cells/mL and 1.1 × 10^9^ cells/mL for OD_600 nm_ 1.6 and 2.5, respectively, for WB and WS substrates). Protein abundance was correlated to cell growth (Figure 2). A maximal yield was obtained with glucose as a substrate (1972 ± 172 μg/mL), whereas the culture grown on WB produced only 739 ± 53 μg/mL of proteins). As *T. xylanilyticus* growths are not identical in the case of the four substrates tested, direct protein concentration comparisons are not relevant. We normalized protein concentration values with cultures absorbencies. Thus, WB yielded better protein production (560 ± 40 μg/DO_600 nm_). Xylan and WS gave equivalent protein yields (316 ± 32 μg/DO_600 nm_ and 301 ± 23 μg/DO_600 nm_, respectively). Concerning protein localization, a significant increase of excreted proteins was observed in case of culture on xylan, WB and WS (data not shown).

### Effect of lignocellulosic composition on *T. xylanilyticus* growth

WB and WS substrates contained different amounts of carbohydrates (74% and 78% of the dry matter, respectively). A high content of arabinoxylan was found in WB (51% of the dry matter) compared to WS (33% of the dry matter). The ratios of arabinose and xylose residues varied between the two plant materials from 0.72 to 0.14 in WB and WS, respectively (data not shown). Because the aim of this study was to evaluate the impact that the chemical composition of the lignocellulosic biomass had on the growth of *T. xylanilyticus*, residual carbohydrates, phenolic and acetyl ester contents were analyzed after 24 h, 48 h and 72 h in successive cultures grown on the same plant cell wall materials. Cell densities in the supernatant phase were also monitored. As shown in Table 1, the carbohydrate contents of WS and WB decreased to 67% and 72%, respectively, after 24 h of growth. The decrease observed in WB was less than that observed in WS; however, considering the loss of dry matter during culture growth, which was important for WB (39% during the first 24 h), the decrease in sugar content represented 54% of the total loss in WB vs. 20% in WS. In accordance with data on total carbohydrates, xylose and glucose contents were also altered. Taking into account the loss of dry matter, xylan loss represented 64% and 15% of the loss for WB and WS, respectively, during 24 h of growth. *T. xylanilyticus* used approximately 26% and 39% of the glucan fraction during 24 h growth on straw and bran, respectively. Quantification of phenolic ester linked molecules showed a dramatic decrease in ferulic, *p-*coumaric and diferulic acid contents during growth on bran, whereas in the case of straw none of these modifications was observed. Given the residue yields remaining after growth, the loss of ferulic, *p*-coumaric and diferulic acids represented 89%, 86% and 73% of the total loss, respectively, for WB and only 16%, 15% and 21% of the total loss, respectively, for WS. During growth on WB, 33% of acetyl ester linkages were cleaved after 24 h vs. only 10% for WS. In parallel, *T. xylanilyticus* grew very well during the first 24 h as cell concentrations reached 1.0 × 10^9^ cfu/mL for both substrates. During the second and the third cultures, no significant changes were observed in the chemical composition of the residues. The loss of total carbohydrates was 10% for both substrates after 48 h of growth. This result represented a loss of xylose and glucose in WS and a loss of primarily arabinose in WB. In total, 30% and 5% of the acetyl content was liberated from WS and WB, respectively. Despite this minimal change in chemical composition, growth remained significant (1.0 × 10^9^ cfu/mL and 7.4 × 10^8^ cfu/mL on WB and WS substrates, respectively). After 72 h in culture, growth decreased drastically as cell densities did not increase beyond 2.7 × 10^6^ cfu/mL on WB and 1.7 × 10^8^ cfu/mL on WS. No significant chemical modifications occurred in the residues after 72 h of growth.

**Table 1:** **Chemical compositions of plant cell walls after *T. xylanilyticus *growth**

**WS residues**	**Yield (%)^a^**	**Total sugars (%)^b^**	**Ara (%)^b^**	**Xyl (%)^b^**	**G (%)^b^**	**Cell density cfu/mL^c^**	**Phenolic esters^d^ (mg/g)^e^**			**Acetyl esters (mg/g)^e^**
							**pC**	**Fe**	**DiFe**	
0 h	86	78	4	28	44	0.0	5.7 ± 0.3	3.2 ± 0.1	0.3 ± 0.0	31.0 ± 0.1
24 h	80	67	4	26	35	2.5	5.2 ± 0.4	2.9 ± 0.1	0.3 ± 0.0	29.0 ± 0.2
48 h	73	66	4	26	35	1.9	5.7 ± 0.3	3.3 ± 0.3	0.3 ± 0.0	23.0 ± 1.2
72 h	76	63	4	27	32	0.6	5.5 ± 0.2	3.0 ± 0.1	0.3 ± 0.0	26.1 ± 4.3
**WB residues**	**Yield (%)^a^**	**Total sugars (%)^b^**	**Ara (%)^b^**	**Xyl (%)^b^**	**G (%)^b^**	**Cell density cfu/mL^c^**	**Phenolic esters^d^ (mg/g)^e^**			**Acetyl esters (mg/g)^e^**
							**pC**	**Fe**	**DiFe**	
0 h	84	74	21	31	22	0.0	0.2 ± 0.0	6.8 ± 0.1	0.9 ± 0.0	8 ± 0.9
24 h	45	72	23	22	25	2.7	0.0 ± 0.0	1.4 ± 0.1	0.5 ± 0.0	8.9 ± 0.1
48 h	48	69	23	21	24	2.6	0.0 ± 0.0	1.4 ± 0.1	0.5 ± 0.0	9 ± 0.0
72 h	45	75	24	21	27	0.2	0.1 ± 0.0	1.3 ± 0.2	0.6 ± 0.1	9.8 ± 0.4

^a^: total dry matter; ^b^: Carbohydrates are given as % of total dry matter; ^c^: Cell density is given as 10^9^ cfu/mL; ^d^: Phenolic acid represents ferulic, p-coumaric and total diferulic acids; ^e^: Ester contents are given as mg per g of residues.

### Enzyme production analysis

**Figure 3:**
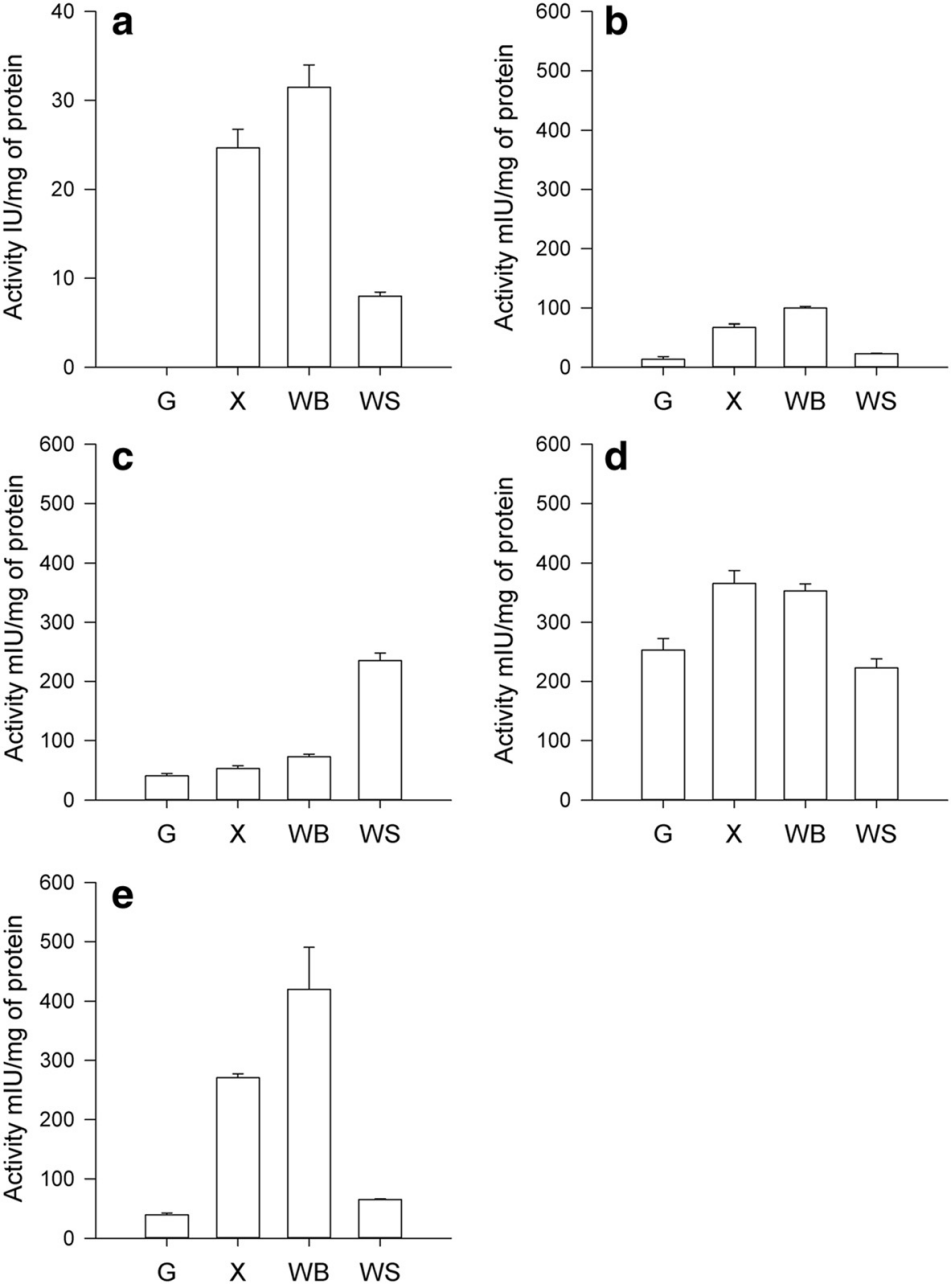
**Hemicellulolytic enzyme production by *T. xylanilyticus *on various substrates during the stationary phase.** (**a**) endoxylanase activity; (**b**) xylosidase activity; (**c**) feruloyl-esterase activity; (**d**) acetyl esterase activity; and (**e**) arabinofuranosidase activity. Values are the means of samples performed in triplicate with standard deviation. Activities are expressed as IU/mg of protein or as mIU/mg of protein and represent the sum of extracellular, cytoplasmic and membrane-associated activities. G, glucose; X, xylan; WB, wheat bran; and WS, wheat straw.

Endoxylanase activities were observed in the different protein fractions of *T. xylanilyticus* when the bacterium was cultivated on xylan, WB and WS. As expected, no xylanase activity was measured in the cultures grown on glucose. Xylanase activity represented the primary category of enzymatic activity produced by *T. xylanilyticus* on xylans, WB and WS. The yields obtained were significantly higher on WB cultures (31.5 ± 4.3 IU/mg) compared to xylan and WS cultures (24.7 ± 2.3 IU/mg and 8 ± 0.46 IU/mg, respectively; P < 0.01 and P < 0.007, respectively) (Figure 3a). As shown in Table 2, xylanases were always highly produced extracellularly, with 86.5%, 82% and 73% of the total xylanase activity resulting from extracellular production on xylan, WB and WS, respectively. An increase in cytosoluble xylanase activity was observed during growth on lignocellulosic biomass (16% and 21% on WB and WS, respectively, compared to 7% on xylan).

**Table 2:** **Repartition of enzymatic activities produced by *T. xylanilyticus *grown on various substrates**

**Xylanase (IU/mg)^a^**				**Xylosidase (mIU/mg)^a^**		
**Localization**	**Extracellular**	**Cytoplasmic**	**Membrane associated**	**Extracellular**	**Cytoplasmic**	**Membrane associated**
**glucose**	nd	nd	nd	2.2 ± 0.6	11.4 ± 3.8	nd
**xylan**	21.4 ± 1.6	4.7 ± 0.1	1.6 ± 0.1	4.7 ± 0.6	62.7 ± 5.0	nd
**wheat bran**	25.9 ± 3.5	4.9 ± 0.8	0.6 ± 0.0	3.1 ± 0.3	96.6 ± 2.5	nd
**wheat straw**	5.8 ± 0.3	1.7 ±0.2	0.5 ± 0.0	4.2 ± 0.3	18.9 ± 1.0	nd
**Feruloyl-esterase (mIU/mg)^a^**				**Acetyl-esterase (mIU/mg)^a^**		
**Localization**	**Extracellular**	**Cytoplasmic**	**Membrane associated**	**Extracellular**	**Cytoplasmic**	**Membrane associated**
**glucose**	12.0 ± 0.8	27.4 ± 1.0	1.5 ± 0.7	109.6 ± 4.6	174.7 ± 13.3	26.6 ± 1.9
**xylan**	5.8 ± 0.6	23.9 ± 1.1	23.8 ± 2.9	76.2 ± 8.0	289.5 ± 13.0	nd
**wheat bran**	15.9 ± 2.2	14.3 ± 1.4	43.09 ± 0.7	64.2 ± 5.8	288.8 ± 35.3	nd
**wheat straw**	15.9 ± 2.7	146.4 ± 2.8	73.1 ± 4.3	130.0 ± 0.7	93.0 ± 14.5	nd
**Arabinofuranosidase (mIU/mg)^a^**						
**Localization**	**Extracellular**	**Cytoplasmic**	**Membrane associated**			
**glucose**	6.7 ± 0.3	32.2 ± 2.7	1.0 ± 0.0			
**xylan**	16.0 ± 1.0	230.8 ± 2.2	23.9 ± 3.5			
**wheat bran**	17.5 ± 0.2	399.1 ± 27.5	3.51 ± 0.6			
**wheat straw**	0.6 ± 0.0	64.3 ± 1.2	0.7 ± 0.1			

^a^ Activities are given in IU per mg of protein for xylanase and in mIU per mg of protein for arabinofuranosidase, feruloyl, acetyl-esterase and xylosidase activities nd: not detected.

Xylosidase activity corresponded to enzymes implicated in the release of monomeric xylose from xylo-oligosides produced by endo-enzymes. Xylosidase activity ranged between 13.6 ± 4.5 mIU/mg (for glucose) and 99.7 ± 2.8 mIU/mg (for WB). Compared to glucose, the complex substrates induced greater production of xylosidase. The maximum yield was obtained with WB (a 7-fold increase compared to the glucose level), whereas increases were approximately 1.7- and 5-fold on xylan and WS substrates, respectively (Figure 3b). More than 90% of xylosidase activities were found in the soluble protein fraction of *T. xylanilyticus* (Table 2).

Debranching enzymes include enzymatic proteins that are able to remove lateral ramifications from xylan chains. Feruloyl esterase activities were detected in all cultures of *T. xylanilyticus* (Figure 3c). Measured activities were approximately 41.0 ± 3.9 mIU/mg (glucose) and 235.3 ± 12.8 mIU/mg (WS). Feruloyl esterase activity was also induced in *T. xylanilyticus* grown in the presence of complex substrates, as increases of 1.4-, 1.8- and 5.7-fold were observed on xylan, WB and WS substrates, respectively, compared to glucose (P <0.01; P <0.01; P <0.001). The activities were primarily membrane-associated or occurred in the soluble protein fractions. On xylan and WB, the induction effect was mediated by an increase in membrane-associated activities, while on WS, both soluble and membrane-associated activity levels rose strongly. This led to higher amounts of feruloyl esterase activity on WS (Table 2).

Acetyl esterases were detected in the intracellular fractions of *T. xylanilyticus* proteins (Figure 3d). Intracellular activities represented 56%, 79% and 82% of total activities on glucose, xylan and WB substrate, respectively. Surprisingly in case of WS, the proportion of activities in the excreted fractions was comparable to the proportion of activities in the intracellular fractions (58% and 42%, respectively). Compared to glucose, an induction of 1.66-fold was observed in intracellular acetyl esterase on xylan and WB (P<0.01). No significant induction of total acetyl esterase activity was observed with WS; however, an increase of 2.5-fold was detected in excreted acetyl esterase (P<0.001). The highest activity levels obtained were 365.7 ± 21.8 mUI/mg and 353.0 ± 41.4 mUI/mg on xylan and WB cultures, respectively.

Arabinofuranosidase activities were detected in all cultures of *T. xylanilyticus* (Figure 3e). These activities were essentially intracellular, as more than 80% of the activity was detected in the soluble protein fraction of *T. xylanilyticus* (Table 2). Arabinofuranosidase activity was approximately 40.0 ± 3.0 mIU/mg in cultures grown on glucose. An induction effect was observed when *T. xylanilyticus* was grown on complex substrates, with 7- and 1.6-fold increases observed for xylan and WS, respectively (P < 0.001 and P < 0.01). The maximum induction effect was obtained with WB, with arabinofuranosidase activity quantified at approximately 420.1 ± 70.0 mIU/mg. This result represented an increase of 10-fold compared to activity measured in the glucose culture and more than 1.5-fold compared to activity measured in the xylan culture (P < 0.001 and P < 0.01).

### Enzymatic strategies to fractionate bran and straw

To further examine the activity panels produced by the various substrates and to investigate the mechanism by which *T. xylanilyticus* fractionates a complex biomass, percentages of each activity obtained on xylan, WB and WS were compared. If 100% of production represented the sum of all activities, endoxylanase activity represented the primary hemicellulolytic activity produced by the bacterial cells. Endoxylanase activity represented 97% of the total enzymatic activity in cultures grown on xylan and WB. For WS cultures, the amount of xylanase dropped significantly, to 93.6% of the total activity. This decrease was inversely correlated with the increase in exo-enzyme activity, with a maximal yield obtained in the WS culture (6.4% of total production). A focus on accessory enzyme production showed that on WB, arabinofuranosidase levels increased compared to the WS and xylan cultures (1.3% vs. 1.1% and 0.8%, respectively), whereas on WS, feruloyl and acetyl esterase activities represented the largest proportion (5.4% of the total activities) of exo-enzyme activities. Xylosidase activity was determined to be similar under all three substrate cultivation conditions.

## Discussion

### Growth on lignocellulosic substrates and modulation by residue composition

To improve our understanding of feedstock biomass deconstruction by the hemicellulolytic bacterium *T. xylanilyticus*, its ability to use destarched WB and WS was studied and compared to its growth on simple substrates (xylan and glucose). Multiple biological assays were performed with the aim of providing insight into the physiological behaviors of the bacteria on various substrates. Our results confirm previous studies that indicated that *T. xylanilyticus* uses glucose and xylan as its primary carbon sources with equal efficiency [16,17,19]. Similar trends were obtained for the hemicellulolytic bacterium *Paenibacillus* JDR-2, which utilizes methyl-glucurono-arabinoxylans as well as glucose or xylose, [27]. In the case of cellulolytic anaerobes such as *Clostridium phytofermentans* and *Caldicellulosiruptor saccharolyticus*, growth on hemicellulosic substrates is more efficient than growth on monomeric substrates [28,29]. We showed that *T. xylanilyticus* is able to use WB and WS biomass efficiently, as cell densities on this biomass were greater than 10^8^ cells/mL during the stationary phase. Both cultures exhibited high growth rates and few sporulation. Yang, et al. showed that *Anaerocellum thermophilum* was able to use plant cell walls and its growth was similar on switchgrass and poplar [30]. *C. saccharolyticus* utilized complex carbohydrates contained in acid-pretreated switchgrass and poplar, however, the doubling time was significantly higher on the lignocellulosic biomass compared to xylose or glucose. Indeed, the doubling time on poplar was twofold higher than the doubling time on pretreated switchgrass, suggesting better utilization of the latter [29]. *Caldicellulosiruptor obsidiansis* was also able to use acid-diluted pretreated switchgrass, but its growth rate was significantly lower compared to growth on cellobiose [31]. Concerning sporulation which occurred later in case of cultures in presence of lignocellulosic biomass, it is possible that the higher cellular biomass produced on glucose and xylan led to a rapid use of substrates and then to sporulation. On complex substrates, several authors observed an accumulation of reducing sugars during stationary phase growth indicating that what is produced during extracellular degradation may not be assimilated directly [27,32]. One could suppose that during culture of *T. xylanilyticus* on lignocelluloses, soluble sugars could be potentially available during a long time and support a slower growth at the stationnary phase and then delay the sporulation step. In our study, growth profiles obtained on WB and WS were different as WB was used more efficiently than WS by *T. xylanilyticus.* On WS, the doubling time was significantly higher compared with WB, and the growth kinetics indicated the presence of a double growth. It is possible that an adaptation phase was needed on WS as the inoculum was produced with xylan cultures. During this phase *T. xylanilyticus* cells used soluble compounds in the medium (yeast extract or soluble sugars) to grow. When those substrates are exhausted, the second lag phase occurred. Xylans in WS are not very accessible; bacteria could induce the production of hydrolytic enzymes which are necessary for the fractionation of recalcitrant substrate allowing then the second growth. This latter one was more efficient. The difference of accessibility and then difference of growth on straw and bran could be explained by the different compositions of both lignocellulosic substrates. The low lignin content in WB (3.4%) renders its xylans and glucans more accessible to *T. xylanilyticus*[33]. A longer adaptation phase was needed in case of WS which contains high levels of lignin (20% of the total dry matter) [34]. Analysis of residue compositions after the cultures were grown indicated that the bacteria were able to use approximately 50% of the total carbohydrates present in WB after 24 h, whereas the bacteria used only 20% of the total carbohydrates in WS. An increase in the cultivation time (to 48 h and 72 h) did not improve the utilization rates of carbohydrates and growth stopped after 48 h in the cultures. This result suggests that the quantity of non accessible residual carbohydrates to *T. xylanilyticus* is more important in WS than in WB. The responsiveness to hydrolysis and conversion of WS could probably be improved by low severity pretreatments such as hot water or ammonia.

### Global protein and enzyme production

Considering the complexity of lignocellulosic feedstocks, the fractionation of these complex environments represents a difficult challenge for microorganisms. However, few studies have focused on the correlation between growth, microbial physiology and enzyme production. In our study, we hypothesized that with plant cell walls as the primary carbon source, bacterial growth could be correlated with enzyme production because the enzymes produced are needed to liberate monomeric sugars that support biomass production. During this study, we observed that *T. xylanilyticus* was able to produce large quantities of proteins when cultivated on glucose. This substrate allows for the production of large amounts of biomass, even if the associated enzymatic activities are low. On a more complex substrate (xylan or lignocellulose), the amounts of biomass obtained were lower; however, protein production was higher, particularly the production of excreted and enzymatic proteins. This effect could be because the majority of bacterial hemicellulolytic systems are inducible and are controlled by catabolic repression and carbon availability. Such systems enable the microorganism to adapt rapidly to difficult environments or to complex substrates, which prevents competition with other organisms [35-37]. *T. xylanilyticus* is able to adapt its protein production in response to the presence of xylan or more complex substrates. This adaptation results in the production of a large quantity of hemicellulolytic enzymes. Cultivation on WB allows for better global enzyme production, in particular a high level of endoxylanase production. Global enzymatic activities are lower on WS, and this finding is correlated with lower endoxylanase production. One explanation could be that in WS cultures, optimal xylanase production does not occur in the stationary phase. Xylanase, debranching enzyme and xylosidase activities were detected when *T. xylanilyticus* was grown on complex substrates, suggesting cooperative action between endo-enzymes and exo-enzymes. However, xylanase activity was primarily found in the excreted partition or/and cell associated, whereas debranching enzyme and xylosidase activities were cell associated or were found in the intracellular fraction. This repartition was observed earlier in other hemicellulolytic and cellulolytic bacteria. The secretion of a primary endo-enzyme in the culture medium allowed the oligomerization of polysaccharides into substituted oligosaccharides which can enter in the cells and are further processed by membrane or intracellular exo-acting and debranching enzymes. These mechanisms suggest an ecological strategy employed by these bacteria that prevents the end products from becoming available to other bacteria. The proximity of resulting hydrolysis products would decrease diffusion dependant assimilation rates [28,38].

Enzyme production represents a limiting step for lignocellulosic fractionation. Various studies have shown that using the cellulolytic and hemicellulolytic secretomes of fungi improves enzyme cocktails [39,40]. The use of bacterial and thermophilic enzymes may present many advantages, as proteins are often thermostable and resistant to large ranges of pH [41]. Moreover, the use of fungal secretomes could under-represent enzymatic activities, as lignocellulolytic activities can be intracellular, cell-associated or extra-cellular. Due to different experimental conditions, it is difficult to compare our results with those of previous studies. However, it is interesting to note that under our experimental conditions, the endoxylanase and auxiliary enzyme production obtained from *T. xylanilyticus* was comparable to or greater than that obtained with the various fungal crude commercial enzymes evaluated by Chundawat et al. [39].

### Strategies used by *T. xylanilyticus* to fractionate plant cell walls

To provide insight into the biomass degradation strategy used by *T. xylanilyticus*, our second hypothesis was that on various complex substrates, microorganisms could produce adaptable enzymes that can more efficiently fractionate their growth substrates and that changes in enzymatic activity can explain how bacteria attack plant cell materials. In the literature, most studies have focused on using proteomic analyses to identify global changes in protein profiles to assess growth on various substrates or on evaluating endoxylanase production, and studies focusing on the correlations between enzymatic activities have been rare [31,42]. To verify our hypotheses, we chose to estimate endoxylanase, debranching enzyme and xylosidase activities during the stationary growth phase of *T. xylanilyticus*. Endoxylanases are the primary enzymes implicated in hemicellulose conversion by *T. xylanilyticus*. However, auxiliary enzyme production profiles differed depending on the growth substrate. WB and WS did not induce the same enzymatic activities. On WB, the arabinofuranosidase activity was higher, it is probably necessary to remove the arabinose residues present in large quantities in this substrate (Ara/Xyl = 0.72). Because, WB cannot have access to the inner part of the bacterial cell in which arabinofuranosidase activities were produced, it is possible that induction was due to the presence of soluble oligosaccharides (substituted or not with arabinose) which are produced by xylanases and could be internalized into the bacterial cells. Higher production of arabinofuranosidase was accompanied by a large decrease in arabinose content in bran during the first 24 h of growth. Arabinose liberation could support bacterial growth after 24 h, when xylose and glucose are not available. Induction of arabinofuranosidase activity was also observed in *Thermobifida fusca* grown on cellulose and lignin [42], indicating that these activities seem to be important for the hydrolysis of lignocellulosic plant cell walls in which these polymers are embedded with arabinoxylans. Arabinofuranosidase activity could allow xylanases better access to the xylan components of WB.

Interestingly, when *T. xylanilyticus* grew on WS, the proportion of esterase activity was higher compared to glucose and other substrates. Both cytoplasmic and extracellular feruloyl esterase activities but also extracellular acetyl xylan esterase activities were induced. This is associated with a high content of acetate and phenolic acids (especially *p*-coumaric acid) into WS substrate which could induce more feruloyl-esterase enzyme production. On WB and xylan, feruloyl esterase activity associated with membranes were induced but in a lesser extent, whereas intracellular acetyl xylan esterase activity increased noticeably. One can conclude that the esterases implicated in WB fractionation are not the same as those implicated in WS degradation. Recent studies have shown that three feruloyl esterases are produced by the hemicellulolytic rumen bacteria *Cellulosilyticum ruminicola* H1 [43]. These enzymes differ in substrate specificity and in cellular localization and had different working patterns when they were associated with xylanases and cellulases. The authors suggested that they could play a distinct role in lignocellulose degradation in the rumen. Similar results for acetyl xylan esterase activities were observed in fungi [44]. Adav et al. showed for the first time that acetyl xylan esterase is upregulated in the presence of lignin [42]. It is possible that the high content of lignin in straw residues could induce high levels of esterase production by *T. xylanilyticus*. The esterase activities produced are implicated either in the cleavage of the feruloyl groups that link xylans to lignins or link two xylans chains together via diferulic bridges or in the cleavage of ester bonds between acetyl residues and xylose residues during the first 24 h of growth.

On WB, high levels of hemicellulolytic enzymes were obtained. The hemicellulolytic enzyme panels were characterized by the presence of significantly higher arabinofuranosidase levels. Lower growth and enzymatic production in bacterial cultures grown on WS could be due to the high quantity of lignin, which could hinder the hemicellulose fraction and impede the action of the main hemicellulolytic enzymes. However, this limitation was overcome by the bacterial production of high levels of accessory enzymes such as esterases.

As WB and WS are too large to pass through the bacterial membrane, small oligosaccharides released from depolymerization of WS and WB by extracellular or membrane associated xylanases could act as inducers of cytoplasmic, membrane associated accessory enzymes or the primary xylanase production observed in *T. xylanilyticus* cultures. Several studies showed the implication of xylobiose, xylotriose and methylglucuronoxylotriose in the induction of hemicellulolytic enzymes [35,38]. Since WB and WS had very dissimilar compositions, enzymes profile production and enzymatic localization, it is possible that inducers are not the same in both cases. The details of this induction mechanism remain to be elucidated.

## Conclusion

An important bottleneck in lignocellulose fractionation is the ability to obtain functional biocatalysts and to qualitatively and quantitatively define the enzymes necessary for an optimal balance between biomass deconstruction and enzyme costs. Here, we showed that *T. xylanilyticus* actively grows on plant cell walls and produces hemicellulolytic enzymes. These enzymes are robust, thermostable and able to efficiently fractionate plant cell walls [22,25]. It is clear that the bacterium can adapt its enzymatic profile to better address the composition of various lignocellulosic substrates. *T. xylanilyticus* employed concerted enzymatic strategies to grow and to degrade the hemicellulolytic portion of plant cell walls. Part of its strategy is the utilization of debranching enzymes. These results are interesting because they provide evidence for the development of functional enzymatic cocktails that allow for better fractionation of the hemicellulose portion of plant cell walls and better access to the cellulosic portions of the plant. We conclude that *T. xylanilyticus* presents interesting advantages that make it a good model for studying physiological approaches to enzyme production and lignocellulose degradation, with the aim of developing the key enzymes.

## Methods

### Strain and media

*Thermobacillus xylanilyticus* strain XE, isolated from a manure heap, was used in this study. The strain was available at the Collection Nationale de Cultures de Microorganismes (France) under the number CNCM I-1017. The bacterial strain was cultivated on basal medium supplemented with 10% CO_2_, as described by [17]. For growth kinetics, glucose (Sigma Aldrich, France) solution and destarched wheat bran and wheat straw (Apache variety, 2 mm) provided by ARD (Pomacle, France) were autoclaved separately and then added to autoclaved basal medium at a concentration of 5 g L^-1^ in 1 L bottles. Oat spelt xylan (Sigma Aldrich, France) was autoclaved together with the basal medium at a concentration of 5 g L^-1^.

### Growth kinetics on glucose, xylans, wheat straw and bran

Cultures were inoculated with 1% (v/v) of non-sporulated preculture (OD _600 nm_ = 2 on oat spelt xylan medium) and incubated at 50°C and 130 rpm with glucose, xylan, bran and straw (as primary carbon sources) in 1 L bottles. Growth was tracked by monitoring light scattering at 600 nm with a Uvikon 933 spectrophotometer over a period of 24 h. Absorbencies were read directly or after briefly harvesting wheat bran and straw particles by centrifugation for 30 sec at 500 × g. Samples of culture media containing the various carbon sources were incubated under the same conditions without inoculation, and these samples were used as blanks for reading absorbencies. Lag, acceleration, exponential and stationary phases were determined on a graph representing Ln(OD _600 nm_) = f(t). Doubling times (d) were calculated during the exponential phase according to the formula: n = (Ln(ODt_2_) – Ln(ODt_1_))/Ln(2) and d = t_2_-t_1_/n where n represents the number of generations. Kinetics experiments were performed in triplicate, and the means of the doubling time and the standard deviation were calculated.

Cell counts were made on a Neubauer cell counter using a phase contrast microscope (Nikon 1528, Japan) with 1000 × magnification, and by inoculating basal-glucose medium solidified in 15 g agar L^-1^ with 100 μL of diluted liquid culture (10^-6^ to 10^-8^). Cellular concentration was expressed as cells per mL.

### Successive growth on straw and bran and residue composition

**Figure 4:**
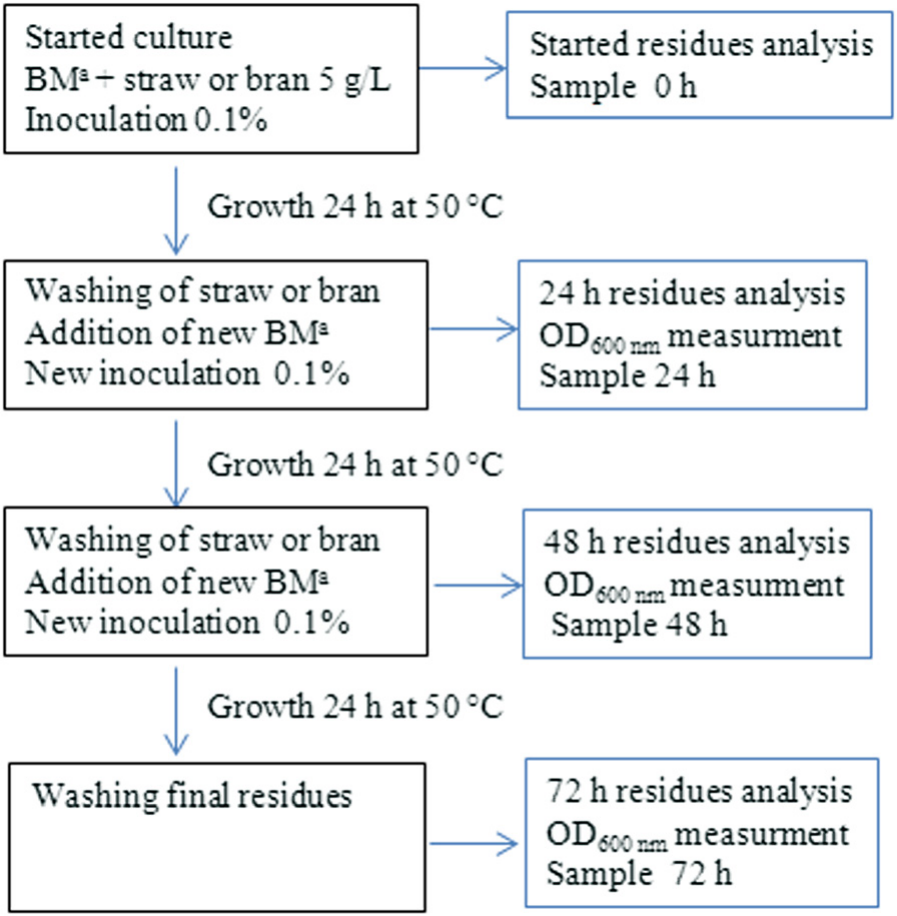
**Scheme for successive *T. xylanilyticus *cultivation on wheat bran and straw.**^a^Basal medium.

To evaluate the limitations of cell growth on bran and straw, successive cultivations with these substrates were performed as described in Figure 4. After the first culture (24 h) on WB and WS, cells were removed and bran and straw particles were washed three times with sterilized distilled water before the addition of new basal medium. The reconstituted media were inoculated *de novo* with 1% (v/v) of non-sporulated precultures as described above. Three successive cultures were performed on the same WB and WS materials over a period of 72 h. To evaluate growth, the OD_600 nm_ was measured every 24 h. A negative control (0 h) was prepared by incubating straw and bran with basal medium at 50°C for 24 h without inoculation. Conversion of WS and WB was calculated by comparing the initial sugar contents of the untreated bran and straw with the amount of sugar remaining after 24 h, 48 h and 72 h in presence of *T. xylanilyticus.* For this experiment, sugar content of insoluble substrate was evaluated by HPAEC, after acid hydrolysis as described by [25]. Phenolic acid ester content was evaluated, after alkaline hydrolysis of residues as described by [22]. The acetyl ester content in the alkaline hydrolysis residues was evaluated using an acetic acid kit following the manufacturer’s recommendation (Megazyme, Ireland).

### Crude protein preparation

After growth on the various substrates described, planctonic cells collected during stationary phase in 2 mL vials, were separated to the plant particles by centrifugation for 30 sec at 500 × g, and the three protein fractions were prepared for each condition. Excreted protein fractions were obtained by centrifugating the complete cultured media without plant residues at 10,000 × g for 5 min. The supernatant corresponded to the excreted protein fraction. The pellets of planctonic cells were then resuspended in the same volume of sodium phosphate buffer (NaH_2_PO_4_, pH 7.5), frozen, then sonicated on ice (Vibra-Cell, Bioblock) and centrifuged for 5 min at 10,000 × g. The supernatants contained the intracellular protein fractions. Cell-associated protein fractions were prepared by solubilizing the residual pellets in the same volume of phosphate buffer, pH 7.5, supplemented with 0.2% Triton X-100. Samples were incubated at 4°C overnight and then sonicated. The supernatants, containing the membrane-associated proteins, were separated from the residual pellets by centrifugation for 20 min at 20,000 × g. Protein concentrations were determined by the Bradford method using BSA as a standard [45]. As some soluble proteins could be liberated in the medium from plant residues, protein concentration in the basal medium incubated with the plant residues alone were substracted to the protein concentration of extracellular protein.

### Endo-xylanase activity

Endo-xylanase activity was assayed in triplicate according to a procedure described by [46]. An aliquot of 0.1 mL of the extracted proteins was incubated in 0.9 mL birchwood xylan (Sigma) at 0.5% w/v homogeneously suspended in 50 mM sodium phosphate buffer (pH 7.5). The xylanase test was performed at 50°C for 10 min. The reducing sugars were measured by monitoring absorbance at 420 nm every 2 min and by comparison standard curves describing varying concentrations of xylose. One unit (IU) of enzyme activity was defined as the quantity of enzyme required to liberate one μmol of equivalent xylose per minute at 50°C.

### Arabinofuranosidase and xylosidase activities

Arabinofuranosidase and xylosidase activities were measured in triplicate by determining the rate of hydrolysis of *p*-nitrophenyl α-L-arabinofuranoside (0.5 mM) and *p*-nitrophenyl β-xylopyranoside (0.5 mM) to *p-*nitrophenol. Experiments were performed and measured directly using the absorbance at 401 nm for 5 min at 50 °C with a recording spectrophotometer (Uvikon 933). Reactions were analyzed in buffered conditions (50 mM sodium phosphate buffer, pH 7.5) with a total volume of 1 mL containing 0.1 mL of proteins. The extinction coefficient of *p*NP (ε_*P*NP_) was 15,850 M^-1^.cm^-1^. All substrates were purchased from Sigma Aldrich (France).

### Esterase activities

Feruloyl and acetyl esterase activities were assayed in triplicate against methyl ferulate (Apin Chemicals, UK) and *p*NP-acetate (Sigma Aldrich, France), respectively, as described by [22]. The reaction mixture contained 0.1 mL of fractions containing proteins, 0.1 mL of substrates at 2 mM and 0.8 mL of 50 mM sodium phosphate buffer at a pH of 7.5. The increase in absorbance at 401 nm (corresponding to acetyl esterase) and the decrease in absorbance at 340 nm (corresponding to feruloyl esterase) were measured with a recording spectrophotometer (Uvikon 933) at 50°C for 5 min. The extinction coefficients of ferulic acid and methyl ferulate are 2,532 M^-1^.cm^-1^ and 11,538 M^-1^.cm^-1^, respectively, under the assay conditions.

### Statistics

The results were compared by statistical analysis with Student’s test. Differences were deemed significant at a value of p ≤ 0.05.

## Abbreviations

WB: Wheat bran; WS: Wheat straw; G: Glucose; X: Xylan; Ara: Arabinose; Xyl: Xylose; OD: Optical density; BM: Basal medium.

## Competing interests

The authors declare that they have no competing interests.

## Authors’ contributions

HR carried out successive growth on various substrates and residues composition analysis; conceived and organized the study and drafted the manuscript. BH carried out the enzymes activities measurement and residues composition analysis, NM carried out growth and enzymes production studies. CR helped to analyze results and to draft the manuscript. All authors read and approved the submitted version of manuscript.
